# Restoration of Sagittal Alignment as a Key Factor in Degenerative Spine Surgery: A Comparative Analysis of Outcomes in Postoperative Balanced and Imbalanced Cases

**DOI:** 10.7759/cureus.98975

**Published:** 2025-12-11

**Authors:** Bharat R Dave, Abhijith Anil, Mikeson Panthackel, Ajay Krishnan, Shivanand C Mayi, Ravi Ranjan Rai, Mirant B Dave, Arjit Vashishtha, Amritesh Singh, Mahesh Sagar, Saurabh S Kulkarni, Yogenkumar Adodariya

**Affiliations:** 1 Spine Surgery, Stavya Spine Hospital and Research Institute, Ahmedabad, IND; 2 Orthopedics/Spine Surgery, Pushpagiri Institute of Medical Sciences, Thiruvalla, IND; 3 Spine Surgery, Bhavnagar Institute of Medical Science (BIMS), Bhavnagar, IND; 4 Orthopedics, Geetanjali Medical College and Hospital, Udaipur, IND; 5 Orthopedics, University College of Medical Sciences (UCMS) &amp; Guru Teg Bahadur (GTB) Hospital, Delhi, IND; 6 Orthopedics, Mahatma Gandhi Medical College and Research Institute, Aurangabad, IND

**Keywords:** adult degenerative scoliosis, limited fusions, outcomes, sagittal balance, sagittal imbalance

## Abstract

Introduction

The current consensus for treating sagittal imbalance in degenerative spine disease supports deformity correction. However, there is a growing trend toward limited fusion, particularly in patients presenting predominantly with leg symptoms. The present study aimed to compare radiological and clinical outcomes following lumbar fusion between patients with postoperative sagittal balance and those with residual imbalance.

Methods

This study included 270 patients who underwent limited lumbar arthrodesis and were followed for two years. Radiological parameters-sagittal vertical axis (SVA), pelvic incidence (PI), pelvic tilt (PT), and sacral slope (SS)-were recorded preoperatively and at the final follow-up. Clinical outcomes were assessed using the Oswestry Disability Index (ODI), Visual Analogue Scale (VAS), and Short Form-36 (SF-36). Patients were divided into two groups based on postoperative sagittal alignment: balanced (SVA < 50 mm) and imbalanced (SVA > 50 mm). Both intragroup pre- and postoperative improvements and intergroup differences in outcomes were analyzed.

Results

Out of 270 patients, 194 had good sagittal alignment and 76 had sagittal imbalance (SVA > 50 mm). Both groups showed significant postoperative (two-year follow-up) improvement in quality-of-life scores (VAS, ODI, and SF-36) compared with baseline values (p < 0.001). However, when comparing the degree of improvement between the balanced and imbalanced groups, no statistically significant differences were observed. A significant but weak negative correlation was noted between the change in the VAS score and the socioeconomic status in patients with sagittal imbalance of the spine.

Conclusion

In appropriately selected patients, limited lumbar fusion can achieve favorable short-term outcomes irrespective of postoperative sagittal alignment. These findings suggest that extensive deformity correction may not always be necessary in degenerative cases presenting primarily with leg symptoms.

## Introduction

Aging is inherently a kyphosing process of the spine. The gradual loss of lumbar lordosis (LL) is initially compensated by strategies such as pelvic retroversion (increased pelvic tilt (PT)) and hip flexion. Over time, these mechanisms may fail, leading to decompensated sagittal imbalance. Clinically, this can manifest as mechanical low back pain, forward stooping, and a subjective sensation of falling forward [1].

Multiple studies have highlighted the impact of sagittal balance on health-related quality of life (HRQoL) scores. Several parameters have been proposed to quantify sagittal alignment, with those incorporating pelvic morphology providing better correlation to outcomes [2]. Consequently, classification systems such as that proposed by Berjano and Lamartina advocate deformity correction surgery in patients presenting with sagittal imbalance [3].

However, corrective surgery for adult degenerative scoliosis (ADS) has been associated with complication rates ranging from 16% to 80%. Since 2010, surgical strategies have increasingly focused on limiting the number of fused levels, avoiding pelvic fixation, and minimizing the use of three-column osteotomies. These changes have led to a notable reduction in complication rates [4].

Importantly, normal sagittal balance parameters are variable across populations. While the surgical aim for the sagittal vertical axis (SVA) is <50 mm, Schwab et al. reported a normative range of −20 ± 30 mm, whereas Roussouly et al. calculated a mean of 35 ± 19.4 mm [5,6]. This variability suggests that not all cases with SVA > 50 mm mandate deformity correction surgery. In many patients, sagittal imbalance may be incidental, while the primary complaint (e.g., neurogenic claudication and radiating or instability pain) arises from focal degenerative pathology such as stenosis or instability [7]. Recent evidence has further refined this understanding, shifting focus from strict numerical targets to “functional sagittal balance.” Lafage et al. have highlighted that age-adjusted alignment goals are more clinically relevant than theoretical norms, as elderly patients often recruit compensatory mechanisms effectively [8].

Although studies on the influence of socioeconomic status on clinical outcomes in lumbar fusion surgeries consistently demonstrate that individuals from lower socioeconomic backgrounds exhibit poorer outcomes compared to those from higher socioeconomic strata, it is noteworthy that, in the surgeon’s observation, patients from rural backgrounds-often belonging to the lower socioeconomic spectrum-appear to tolerate deformities comparatively well [9].

The aim of the present study is to compare and evaluate outcomes in patients who underwent limited fusions and have maintained sagittal alignment postoperatively vs. those who have sagittal imbalance postoperatively. Objectives include assessing whether postoperative sagittal alignment influences outcomes of patients and assessing the impact of socioeconomic status on the change of quality of life scores in patients with persistent sagittal imbalance postsurgery. We hypothesize that limited fusion can achieve comparable short-term outcomes regardless of postoperative sagittal alignment.

## Materials and methods

This was a prospective observational study carried out over a duration of three years from August 2018 to August 2021 at Stavya Spine Hospital and Research Institute, Ahmedabad, Gujarat, India. The study was approved by the institutional ethics committee (IEC no. CR/52/Inst/GJ/2013/RR-19) and registered on the Clinical Trials Registry of India (CTRl/2019/08/020540). Written informed consent was obtained from all participants.

A total of 270 patients over the age of 50 years who underwent limited lumbar arthrodesis (up to three disc levels) for degenerative lumbar spine disorders and consented to participate were included. All the cases had predominantly neurogenic claudication and radiating pain symptoms with minimal back pain (VAS < 5). Cases in which a specific cause of back pain, such as segmental instability, could be identified were also included. Cases requiring deformity correction surgery, either for symptomatic deformity or deformity-related back pain, were excluded. Patients with a coronal Cobb angle > 10° were excluded. Patients with heart disease, severe organ insufficiency, or diffuse idiopathic skeletal hyperostosis (DISH) were excluded from the study. Those with active systemic or local infections were also excluded from the study. Persistent pain even while sitting was considered indicative of deformity-related back pain. Patients requiring hand support while sitting due to pain or neurogenic claudication were classified as having significant deformity-related back pain [10]. Those without restriction in sitting time and with a claudication distance of less than 100 m who improved on sitting were considered candidates for limited fusion procedures. Patients requiring walking assistance due to forward stooping or experiencing difficulty in ambulation from progressive forward imbalance were excluded from the study.

The initial clinical evaluation and assessment of HRQoL scores were done in the outpatient department. The HRQoL indices were evaluated in the preoperative period using the Oswestry Disability Index (ODI) [11], VAS for leg pain, and Short Form-36 Health Survey (SF-36) scores. Appropriate permission has been obtained from the relevant sources for the use of the ODI scale. The SF-36 questionnaire consists of eight scales that provide two summary measures: physical and mental health. The physical health measure includes four scales measuring physical function (10 items), physical role (four items), physical pain (two items), and general health (five items). The mental health measure included vitality (four items), social functioning (two items), emotional role (three items), and mental health (five items). Likert scales and yes/no options were used to assess functioning and well-being in this 36-item questionnaire. Evaluation of the radiographs was also done in the same setting. Radiographs were obtained in the free-standing posture with hands placed in the supraclavicular fossa. SVA, pelvic incidence (PI), PT, sacral slope (SS), and LL were measured using Surgimap (version 2.3.2.1) software (Nemaris Inc., Methuen, MA, US). Sagittal parameters were measured independently by two spine surgeons blinded to postoperative outcomes; inter- and intraobserver reliability was ensured by repeating measurements on 20% of radiographs.

The socioeconomic status of each patient was assessed by a trained medical social worker using the modified Kuppuswamy scale [12]. The assessor was not involved in patient care and was therefore blinded to surgical planning and clinical outcomes. Because all assessments were performed by a single trained evaluator, inter-rater reliability was not applicable. The income of the family, educational qualifications, and occupation of the head of the family were used to categorize patients into the upper class, upper middle class, lower middle class, upper lower class, and lower socioeconomic class. A revised version of the original scale, using income levels updated to match inflation to 2019 levels, was used in our study.

The symptomatic levels were identified, and limited fusions were done. Levels were decided according to the classification by Berjano and Lamartina [3]. Depending on the severity of stenosis, number of levels, and complexity of the procedure, either an open procedure or a minimally invasive procedure was opted for. Limited fusion was defined as posterior instrumented fusion involving one to three motion segments, performed using pedicle screws with either posterolateral fusion or minimally invasive transforaminal lumbar interbody fusion (TLIF) depending on stenosis severity and stability. Routine minimally invasive interbody lumbar fusion was performed using tubular retractors.

After undergoing treatment, patients were followed up for a minimum of two years. Although some patients had longer follow-up after surgery, all were assessed at the two-year mark. Repeat standing radiographs were obtained, and HRQoL scores were reassessed. The patients were divided into two groups based on the alignment postoperatively-sagittally balanced group (SVA < 50 mm) and sagittally imbalanced group (SVA > 50 mm). All data were compiled into Microsoft Excel (Microsoft Corp., Redmond, WA, US). The data were checked for normality. All continuous variables were expressed as mean and standard deviation (SD). Qualitative variables were expressed as percentages. For normally distributed data, a parametric test (t-test, Pearson correlation) was used, and for non-normally distributed data, a non-parametric test (Mann-Whitney, Wilcoxon, and Spearman correlation) was used. The level of significance was 5%. For analysis, SPSS version 20.0 (IBM Corp., Armonk, NY, US) was used. Post-treatment HRQoL scores at the two-year follow-up were also compared to assess the impact of treatment on sagittal balance parameters and on the quality of life of patients. In patients with sagittal imbalance (i.e., unbalanced spine), the quality of life was also correlated with the socioeconomic status of the subject. An a priori sample size estimation indicated that a cohort of 270 patients would provide greater than 80% statistical power to detect a minimum clinically meaningful difference of 4 points in the ODI, at a significance level of α = 0.05.

To compare outcomes between sagittally balanced and imbalanced groups, preoperative and postoperative HRQoL parameters, including ODI, VAS, and SF-36 scores, were analyzed. The mean improvement (Δ) for each score was calculated by subtracting postoperative values from preoperative scores. Independent t-tests were then applied to compare the mean change between the groups. Missing data were handled using available-case analysis, with no imputation performed.

## Results

Two hundred seventy consecutive patients undergoing limited instrumented arthrodesis for degenerative lumbar spine disorders were included in the study. The average age of these patients was 62.57 years (±7.26). One hundred eighty-nine (71.6%) of them were women, and 81 (30.4%) of them were men. The median socioeconomic scale category of these patients was 3. Twenty patients were category 1, 87 category 2, 115 category 3, and 49 category 4. Patient characteristics are summarized in Table 1.

**Table 1:** Patient characteristics in our study Values are presented as mean ± standard deviation. The comparisons between postoperative sagittal balance groups (SVA < 50 mm vs. >50 mm) were analyzed using independent sample t-tests. The corresponding t-values and p-values are presented for each parameter. SVA: sagittal vertical axis; PI: pelvic incidence; PT: pelvic tilt; SS: sacral slope; LL: lumbar lordosis

Parameter	Total (N = 270)	Post-op sagittal balance maintained (SVA < 50 mm) (N = 194)	Post-op sagittal imbalance (SVA > 50 mm) (N = 76)	t-value	p-value
Age (years)	70.2 ± 7.2	68.5 ± 6.7	73.4 ± 7.1	-5.11	0.02
Number of levels with only stenosis	2.9 ± 1.1	3.0 ± 1.0	2.7 ± 1.2	1.91	0.34
Number of levels with instability	1.3 ± 0.6	1.2 ± 0.5	1.4 ± 0.7	-2.25	0.29
Number of levels fused	2.2 ± 1.0	2.1 ± 0.9	2.3 ± 1.1	-1.39	0.42
Number of levels decompressed	4.1 ± 1.8	4.0 ± 1.7	4.3 ± 1.9	-1.18	0.37
SVA (mm)	41.2 ± 18.4	25.3 ± 8.4	65.3 ± 21.7	-15.57	<0.001
PI (°)	54.8 ± 10.6	55.1 ± 9.9	54.2 ± 11.7	0.59	0.56
PT (°)	22.3 ± 8.2	19.7 ± 7.3	28.9 ± 8.6	-8.13	<0.001
SS (°)	33.1 ± 9.4	33.9 ± 9.0	31.4 ± 9.8	1.9	0.18
LL (°)	47.8 ± 12.3	51.6 ± 11.0	38.9 ± 12.6	7.61	<0.001

Among the 270 lumbar arthrodesis patients, 194 individuals demonstrated good postoperative sagittal balance with an SVA ≤ 50 mm. These patients showed significant improvement in HRQoL parameters at the two-year follow-up. The ODI improved from an average of 26.00 preoperatively to 5.50 postoperatively. A paired t-test confirmed this change to be statistically significant (p < 0.001). Similarly, the VAS for overall pain showed a marked reduction from 7.20 preoperatively to 0.70 postoperatively (p < 0.001). The SF-36 physical and mental component summary scores also demonstrated significant postoperative improvement with p-values less than 0.001. These findings are summarized in Tables 2, 3.

**Table 2:** Change in SF-36 scores in patients with maintained postoperative sagittal balance Values are presented as mean ± standard deviation. Comparisons between preoperative and postoperative SF-36 scores were performed using paired-sample t-tests. The corresponding t-values and p-values are shown for each component summary. SF-36: Short Form-36 Health Survey; PCS: physical component summary; MCS: mental component summary

SF-36 score		N	Mean	Std. deviation	t-value	p-value
Physical component summary	Pre-op	194	4.75	0.07	41.457	<0.001
	Post-op	194	4.97	0.06		
Mental component summary	Pre-op	194	4.76	0.088	37.629	<0.001
	Post-op	194	4.99	0.056		

**Table 3:** HRQoL indices in patients with maintained sagittal balance Values are presented as mean ± standard deviation. Comparisons between preoperative and postoperative HRQoL indices were performed using paired-sample t-tests. The corresponding t-values and p-values are shown for each parameter. HRQoL: health-related quality of life; ODI: Oswestry Disability Index; VAS: Visual Analogue Scale

HRQoL indices	N	Mean	Std. deviation	t-test	p-value
ODI pre-op	194	26.00	6.92	39.190	<0.001
ODI post-op	194	5.50	6.64		
Overall pain VAS pre-op	194	7.20	1.38	61.243	<0.001
Overall pain VAS post-op	194	0.70	1.13		

Among the 270 lumbar arthrodesis patients, it was noted that 76 patients had an SVA > 50 mm in the postoperative period. However, these patients also showed significant improvement in the HRQoL scores postoperatively at a follow-up of two years.

The ODI improved from an average of 29.5 preoperatively to 8.97 postoperatively. A paired t-test was performed, and this change in the ODI score was found to be significant with a p-value less than 0.001. Similarly, with regard to the VAS score for overall pain, there was a significant decrease from 7.96 preoperatively to 1.66 postoperatively. A paired t-test revealed that the decrease was significant with a p-value less than 0.001. The summary scores of the physical and mental components also showed a statistically significant improvement in the postoperative period at a two-year follow-up, with a p-value of less than 0.001 on application of the paired t-test. These results are summarized in Tables 4, 5.

**Table 4:** Change in SF-36 scores in patients with postoperative sagittal imbalance Values are presented as mean ± standard deviation. Comparisons between preoperative and postoperative SF-36 scores were performed using paired-sample t-tests. The corresponding t-values and p-values are shown for each component summary. SF-36: Short Form-36 Health Survey; PCS: physical component summary; MCS: mental component summary

SF-36 score		Mean	N	Std. deviation	t-value	p-value
Physical component summary	Pre-op	4.67825	76	0.066238	32.227	<0.001
	Post-op	4.90118	76	0.053994		
Mental component summary	Pre-op	4.67358	76	0.096452	24.906	<0.001
	Post-op	4.90597	76	0.051933		

**Table 5:** HRQoL indices in patients with sagittal imbalance Values are presented as mean ± standard deviation. Comparisons between preoperative and postoperative HRQoL indices were performed using paired-sample t-tests. The corresponding t-values and p-values are shown for each parameter. HRQoL: health-related quality of life; ODI: Oswestry Disability Index; VAS: Visual Analogue Scale

HRQoL indices	Mean	N	Std. deviation	t-test	p-value
ODI pre-op	29.50	76	7.24	21.58	<0.001
ODI post-op	8.97	76	7.64		
Overall pain VAS pre-op	7.96	76	1.66	33.813	<0.001
Overall pain VAS post-op	1.66	76	1.33		

The improvement in outcomes between the two groups (sagittally balanced vs. sagittally imbalanced) was compared. Although each group showed statistically significant improvement within itself (p < 0.001), between-group comparisons revealed p-values greater than 0.05 for all parameters. The statistical analysis is shown in Table 6.

**Table 6:** Between-group comparison of improvement (Δ) Values are presented as mean ± standard deviation (SD). Comparisons of improvement (Δ) between patients with maintained and imbalanced postoperative sagittal alignment were analyzed using independent sample t-tests (Welch’s correction applied for unequal variances). The corresponding t-values and p-values are presented for each parameter. Cohen’s d was calculated using pooled SD to estimate effect magnitude. The 95% confidence intervals (CI) for mean differences were derived from the standard errors of the difference in group means. ODI: Oswestry Disability Index; VAS: Visual Analogue Scale; SF-36: Short Form-36 Health Survey; PCS: physical component summary; MCS: mental component summary; Δ: change from preoperative to postoperative value

Parameter	Balanced Δ (mean ± SD)	Imbalanced Δ (mean ± SD)	Mean difference (balanced - imbalanced)	t-value (Welch)	p-value	Cohen’s d	95% CI for mean difference
ODI improvement (pre-post)	20.50 ± 6.90	20.53 ± 7.50	-0.03	-0.030	0.976	–0.004	–2.01 to +1.95
VAS improvement (pre-post)	6.50 ± 1.40	6.30 ± 1.50	0.20	0.989	0.324	0.14	–0.36 to +0.76
SF-36 PCS improvement	0.220 ± 0.07	0.223 ± 0.07	-0.003	-0.304	0.761	–0.04	–0.03 to +0.02
SF-36 MCS improvement	0.230 ± 0.08	0.232 ± 0.07	-0.002	-0.238	0.813	–0.03	–0.04 to +0.03

To look for a correlation between socioeconomic status and patient-reported outcome measures, the Spearman test was used. A significant but weak negative correlation was noted between the change in the VAS score and the socioeconomic status in patients with sagittal imbalance of the spine. There was no correlation between the other HRQoL measures and socioeconomic status in these patients. The results are shown in Table 7.

**Table 7:** Correlation between socioeconomic status and change in HRQoL indices in patients with sagittal imbalance postoperatively Values represent correlation coefficients (Spearman rho) between socioeconomic status and postoperative changes (Δ) in HRQoL indices. Statistical significance was determined using the Spearman rank correlation test (p-values, two-tailed). HRQoL: health-related quality of life; ODI: Oswestry Disability Index; VAS: Visual Analogue Scale; SF-36: Short Form-36 Health Survey

Variables	Spearman rho (correlation coefficient)	p-value (Sig. 2-tailed)
Change in VAS score	–0.355	0.002
Change in ODI score	–0.050	0.670
Change in SF-36 physical component summary	0.153	0.188
Change in SF-36 mental component summary	–0.031	0.791

**Figure 1:**
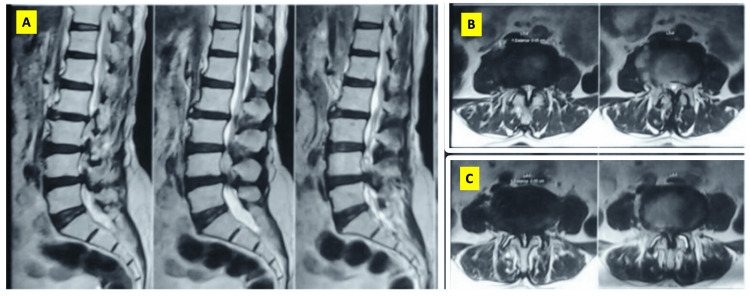
Preoperative MRI images showing L3-L4 and L4-L5 lumbar canal stenosis. Facet effusion noted at the L4-L5 level bilaterally (Patient X) (A) Midsagittal MRI images showing stenosis at the L3-L4 and L4-L5 levels; (B) axial MRI section at the L3-L4 region showing severe stenosis (Schizas C); (C) axial MRI at the L4-L5 segment showing severe stenosis (Schizas D)

**Figure 2:**
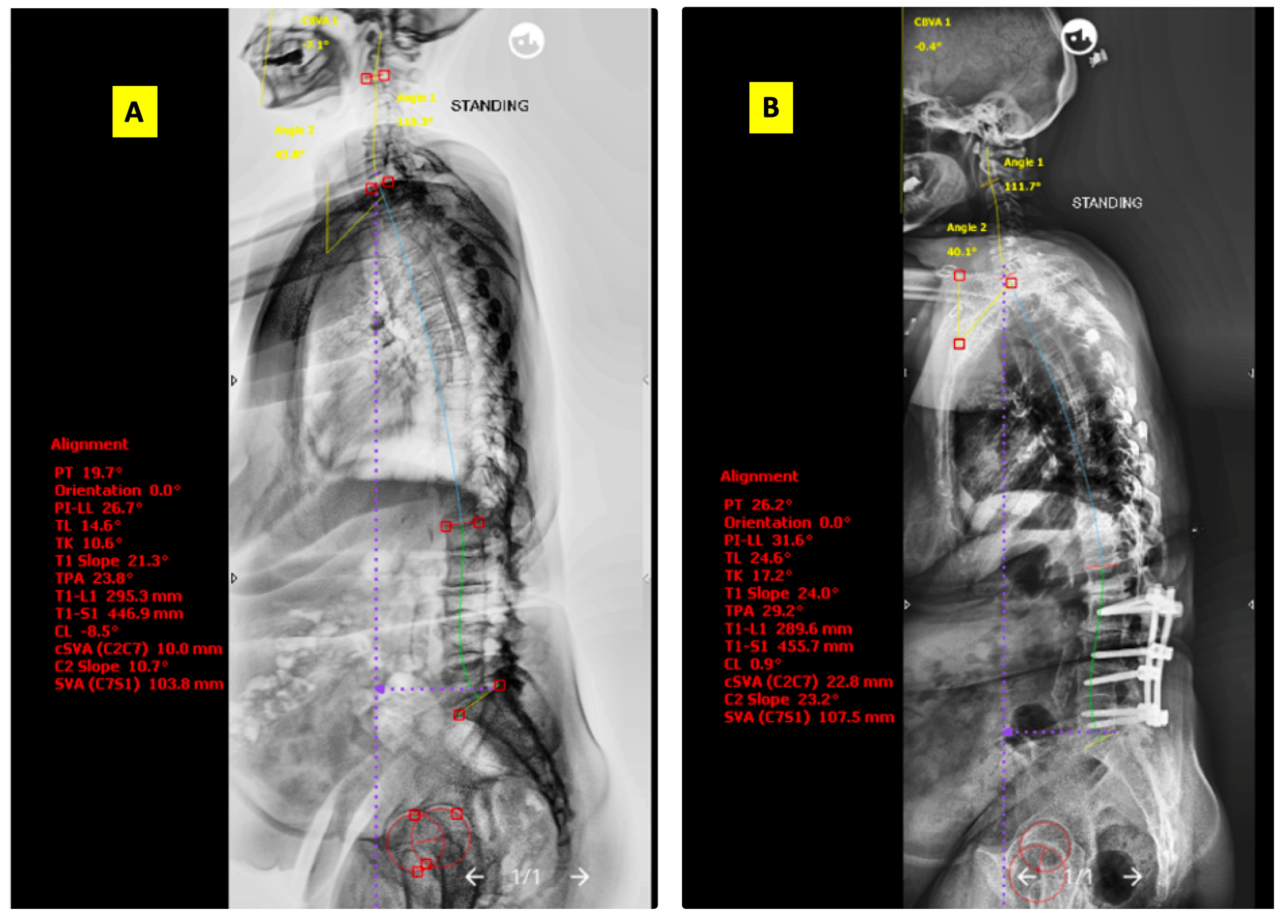
Pre- and postoperative whole spine radiographs with sagittal balance parameters (A) Pre-op standing whole spine radiograph showing SVA 100 mm, suggesting sagittal imbalance. However, she did not have any complaints related to deformity; (B) Post-op standing whole spine radiograph showing SVA 100 mm, suggesting sagittal imbalance not corrected after limited fusion

Given below is a case illustration. Mrs. X, a 63-year-old lady working as a housemaid, presented with major complaints of neurogenic claudication and an instability type of back pain. On examination, she had weakness of the hip abductors. She was finding it difficult to work because of the discomfort in her legs with walking beyond a few steps. On investigation, she was noted to have Schizas grade D lumbar canal stenosis at the L2-L3, L3-L4, and L4-L5 levels with instability at the L2-L3 and L4-L5 levels (Figure 1). Whole spine radiographs showed sagittal imbalance, SVA 103 mm (Figure 2). She was managed with L2 to L5 posterior instrumented decompression and posterolateral fusion. No attempt was made to correct the sagittal imbalance and PI-LL mismatch in view of her advanced age and preoperative anemia.

At the two-year follow-up, she had returned to work and had no limitation in walking. Her VAS score for overall pain had improved from 9 to 1. Her ODI score improved from 82 prior to surgery to 29 postoperatively. This improvement in the HRQoL scores was noted despite not correcting the overall sagittal balance as well as the PI-LL mismatch over the two years.

## Discussion

Two hundred seventy patients over the age of 50 years underwent limited instrumented lumbar spine surgery during the study period. The average age of the patients was 62.57 years. One hundred seventy-nine of them were women, and 79 were men. The median socioeconomic status of our patients was 3, correlating to lower-middle-class patients. One hundred ninety-four had maintained sagittal balance postop while 76 had sagittal imbalance postoperatively.

When comparing preoperative HRQoL measures, patients with sagittal imbalance demonstrated poorer baseline clinical and functional status than those with sagittal balance. Specifically, patients with sagittal imbalance exhibited higher mean ODI scores (29.5 vs. 26.0) and VAS pain scores (7.96 vs. 7.20), indicating greater disability and pain prior to surgery. Their SF-36 physical (4.68 vs. 4.75) and mental (4.67 vs. 4.76) component scores were also marginally lower. This finding may reflect that patients with poorer sagittal balance typically present at a more advanced disease stage and with more severe symptoms.

Although age-related degenerative changes of the lumbar spine are a major contributor to sagittal imbalance, an often underestimated cause is the compensatory lumbar flexion adopted by patients to alleviate neurogenic claudication symptoms. This functional malalignment often improves after decompressive surgery, as the compensatory flexion is no longer required. Studies have reported postoperative sagittal balance correction in approximately 70% of cases. Notably, even among patients with residual imbalance, clinical outcomes were not significantly worse compared with those who achieved balance. This correction has been attributed to the elimination of compensatory lumbar flexion in stenosis cases [13,14]. Thus, in cases undergoing limited fusion procedures, an even greater chance of sagittal balance correction can be anticipated, owing to the lordosing techniques commonly employed during surgery, which help restore physiological lumbar curvature and overall spinal alignment. Hence, in patients presenting with minimal back pain (VAS < 5) and mild, asymptomatic sagittal imbalance, a significant degree of spontaneous postoperative correction can often be expected, even without extensive reconstructive procedures, as decompression alone may restore alignment.

Previous studies evaluating sagittal imbalance and outcomes following decompressive spinal surgery have reported a postoperative sagittal imbalance (SVA > 50 mm) prevalence of approximately 40% among decompressive cases [13]. When comparing clinical outcomes, the difference between sagittally balanced and imbalanced groups was not statistically significant. However, a trend toward better results was observed in the balanced cohort, with good postoperative outcomes noted in 76% of balanced patients, compared to 62% among those with sagittal imbalance [13]. In our study, both the sagittally balanced and imbalanced cohorts demonstrated comparable improvement in patient-reported outcome measures when compared with their respective preoperative scores. Intergroup statistical analysis revealed no significant differences in the magnitude of improvement across HRQoL parameters. These findings suggest that in carefully selected patients with minimal deformity and predominant radicular symptoms, postoperative imbalance may not significantly impact short-term functional outcomes. In cases where deformity-related complaints and back pain are minimal and the main symptoms are due to local compression causing claudication or radiating pain, addressing only the particular levels gives significant functional outcome improvement without the need for deformity correction surgery.

Studies by multiple authors report that postoperative global spine imbalance has a negative impact on the overall quality of life of the patient [7,15-17]. Patients presenting with significant symptoms attributable to sagittal malalignment were excluded from our study. Additionally, we did not include cases with a VAS score greater than 5 for back pain. Considering their lower functional demand, we included only patients over the age of 50 years. Also, our study population consisted of patients from a rural background who were likely to tolerate imbalance and carry on with their activities of daily living. The sustained improvement over a two-year follow-up highlights that the functional gains achieved are not merely short-term. The favorable outcomes observed may, in part, be attributable to robust familial and social support systems, which often differ substantially from those typically encountered in Western patient cohorts.

Patients above 50 years of age often present with multiple comorbidities and poor bone quality, particularly among elderly female patients. In such cases, deformity correction surgery remains a high-risk undertaking. Consequently, management trends in adult degenerative spine disease are shifting toward more conservative approaches such as limited fusion or decompression alone. This paradigm shift is largely driven by the substantial risks associated with deformity correction surgeries; studies have reported complication rates ranging from 16% to 80%. Major risk factors include excessive intraoperative blood loss exceeding 2 L and the presence of three or more medical comorbidities [4,18]. Our findings support limited surgical strategies in elderly or comorbid patients, potentially reducing morbidity without compromising functional outcomes.

We looked for a correlation between the socioeconomic status of the patient and the changes seen in the HRQoL indices. In patients with an SVA > 50 mm postoperatively, a statistically significant negative correlation (Spearman rho 0.355) was noted between the socioeconomic status of patients as per the modified Kuppuswamy scale and the VAS score for pain. This implies that poorer patients tend to tolerate postoperative pain better and are more likely to return to work. No statistically significant correlation was noted between the socioeconomic status of the patient and the changes in other HRQoL parameters.

Our findings imply that in patients with predominant claudication or radiating pain complaints, limited surgical interventions can have equivalent good outcomes, addressing only focal areas that cause neurological symptoms instead of long constructs to restore LL. This increases the scope to perform minimally invasive surgery that can accelerate patient rehabilitation and mobilization, and minimize damage to paraspinal muscles while giving fusion rates equivalent to open surgery.

In view of the conflicting literature, multiple authors have initiated a large multicentric study, the RELApSE study (Registry for Evaluation of Lumbar Arthrodesis Sagittal Alignment), for which a public study protocol has been published in early 2023 [19]. The study aims to enroll 500 patients with a minimum follow-up of five years, with each surgeon contributing between a minimum of 20 and a maximum of 60 patients. The study plans to use a paired t-test to evaluate outcomes among patients with postoperative mismatch between PI and LL using the ODI score and SF-12 scores [19]. Although an increased rate of revision surgery beyond five years has been reported in patients with SVA deviation and PI-LL mismatch in earlier studies, more recent evidence has not demonstrated a significantly higher incidence of revisions due to adjacent segment disease (ASD) in sagittally imbalanced cases [18,20].

The limitations of our study include that this is a single-center study with a relatively short duration of follow-up. Given the existing consensus on the higher incidence of ASD revisions in cases of limited fusion performed in sagittally imbalanced patients [20], a longer follow-up period of at least 5-10 years would be necessary to accurately determine the difference in ASD rates between the two groups. Because the study excluded patients with significant sagittal deformity and those with high baseline VAS back pain, the findings may not be generalizable to all degenerative spine populations. Other limitations include the absence of PI-LL mismatch subgroup analysis, lack of bone mineral density or muscle mass assessment, and the fact that only univariate tests were performed; no multivariate regression modeling was conducted to adjust for potential confounders such as age, comorbidities, or number of fused levels.

## Conclusions

Our findings suggest that limited fusion may achieve satisfactory short-term outcomes even in mild postoperative imbalance; however, restoration of sagittal alignment remains important in selected cases, particularly in younger or more active patients. These observations suggest that limited fusion procedures may yield meaningful clinical benefits in this demographic, obviating the need for long fusion constructs or extensive osteotomies aimed at restoring sagittal alignment. Such strategies are particularly relevant for elderly patients with multiple comorbidities, as they can often adapt through lifestyle modifications even when sagittal imbalance persists. Furthermore, results suggest that patients from lower socioeconomic backgrounds, despite facing economic disadvantages, tend to tolerate postoperative pain more effectively.

## Figures and Tables

**Figure 3:**
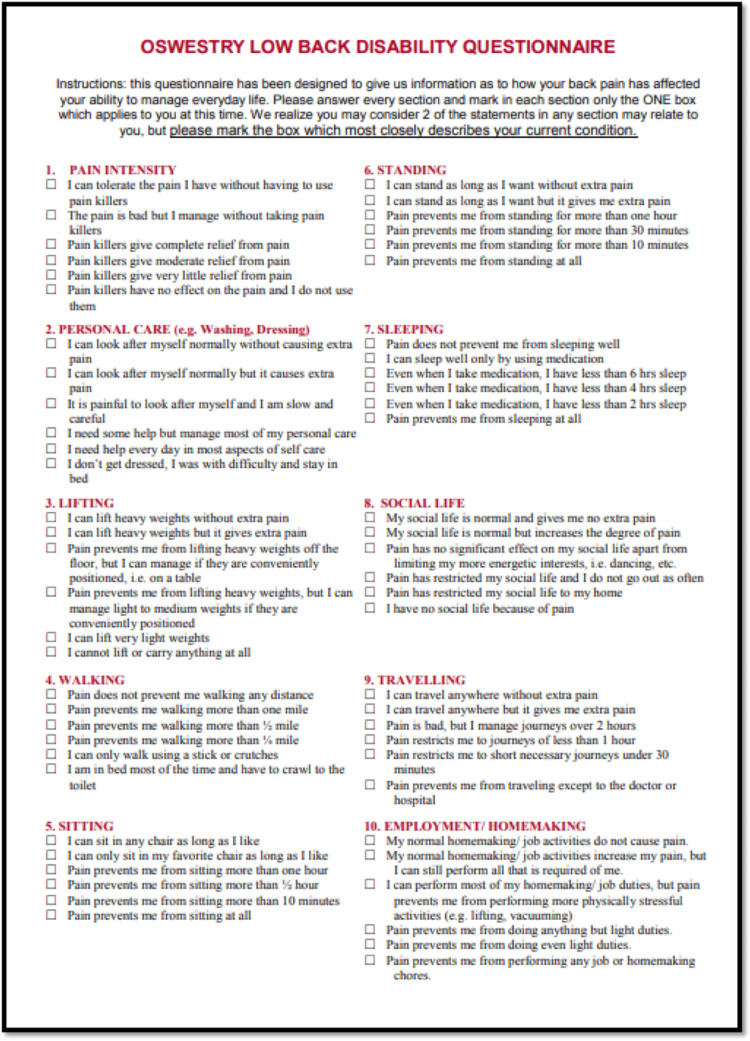
ODI score used in our study ODI © Jeremy Fairbank, 1980. All Rights Reserved. ODI: Oswestry Disability Index
